# Multidimensional Face Representation in a Deep Convolutional Neural Network Reveals the Mechanism Underlying AI Racism

**DOI:** 10.3389/fncom.2021.620281

**Published:** 2021-03-10

**Authors:** Jinhua Tian, Hailun Xie, Siyuan Hu, Jia Liu

**Affiliations:** ^1^Beijing Key Laboratory of Applied Experimental Psychology, Faculty of Psychology, Beijing Normal University, Beijing, China; ^2^Department of Psychology & Tsinghua Laboratory of Brain and Intelligence, Tsinghua University, Beijing, China

**Keywords:** deep convolutional neural network, faces, other race effect, multidimensional face race representation, contact theory

## Abstract

The increasingly popular application of AI runs the risk of amplifying social bias, such as classifying non-white faces as animals. Recent research has largely attributed this bias to the training data implemented. However, the underlying mechanism is poorly understood; therefore, strategies to rectify the bias are unresolved. Here, we examined a typical deep convolutional neural network (DCNN), VGG-Face, which was trained with a face dataset consisting of more white faces than black and Asian faces. The transfer learning result showed significantly better performance in identifying white faces, similar to the well-known social bias in humans, the other-race effect (ORE). To test whether the effect resulted from the imbalance of face images, we retrained the VGG-Face with a dataset containing more Asian faces, and found a reverse ORE that the newly-trained VGG-Face preferred Asian faces over white faces in identification accuracy. Additionally, when the number of Asian faces and white faces were matched in the dataset, the DCNN did not show any bias. To further examine how imbalanced image input led to the ORE, we performed a representational similarity analysis on VGG-Face's activation. We found that when the dataset contained more white faces, the representation of white faces was more distinct, indexed by smaller in-group similarity and larger representational Euclidean distance. That is, white faces were scattered more sparsely in the representational face space of the VGG-Face than the other faces. Importantly, the distinctiveness of faces was positively correlated with identification accuracy, which explained the ORE observed in the VGG-Face. In summary, our study revealed the mechanism underlying the ORE in DCNNs, which provides a novel approach to studying AI ethics. In addition, the face multidimensional representation theory discovered in humans was also applicable to DCNNs, advocating for future studies to apply more cognitive theories to understand DCNNs' behavior.

## Introduction

With enormous progress in artificial intelligence (AI), deep convolutional neural networks (DCNN) have shown extraordinary performance in computer vision, natural language processing, and complex strategy video games. However, the application of DCNNs increases the risk of amplifying social bias (Zou and Schiebinger, 2018). For example, a word-embedding processing system may associate women with homemakers, or a face identification network may match non-white faces to inanimate objects, suggesting the existence of gender and race biases in DCNNs (Bolukbasi et al., 2016). Although the phenomenon of social bias has been widely recognized, the underlying mechanism of such bias is little understood (Caliskan et al., 2017; Garg et al., 2018). In this study, we explored how biased behaviors were generated in DCNNs.

Insight into human biases may help to understand DCNNs' biased responses. A classical race bias, the other race effect (ORE) (Malpass and Kravitz, 1969; Valentine, 1991), shows that people are better at identifying faces of their own race than those of other races (Meissner and Brigham, 2001). The reason underlying the ORE is that people usually have more experiences with faces of their own race (Valentine, 1991), which leads to a better capacity of recognizing faces of their own race. Accordingly, we reasoned that a similar biased response might also be present in DCNNs, as DCNNs tend to perform better on data that most closely resembles the training data. Note that the biased response in DCNNs is not identical to the ORE in humans; however, given the same underlying causes, here we borrowed the term “ORE” to index the biased responses in DCNNs for simplicity. On the other hand, one influential human recognition theory, the face multidimensional representation space (MDS) theory, proposes that ORE comes from the difference in representing faces in a multidimensional space, or simply “face space” (Valentine, 1991; Valentine et al., 2016; O'toole et al., 2018). According to this theory, face space is a Euclidean multidimensional space, with dimensions representing facial features. The distance between two faces in the space indexes their perceptual similarity. Under the frame of this theory, faces of one's own race are scattered widely in the face space (i.e., high distinctiveness) and faces of other races are clustered in a smaller space (i.e., low distinctiveness) (Valentine, 1991; Valentine et al., 2016). Therefore, the higher distinctiveness in representation leads to better recognition of own-race faces than that of other-race faces. In this study, we examine whether the ORE in DCNNs, if observed, may be accounted for by a similar mechanism.

**Figure 1:**
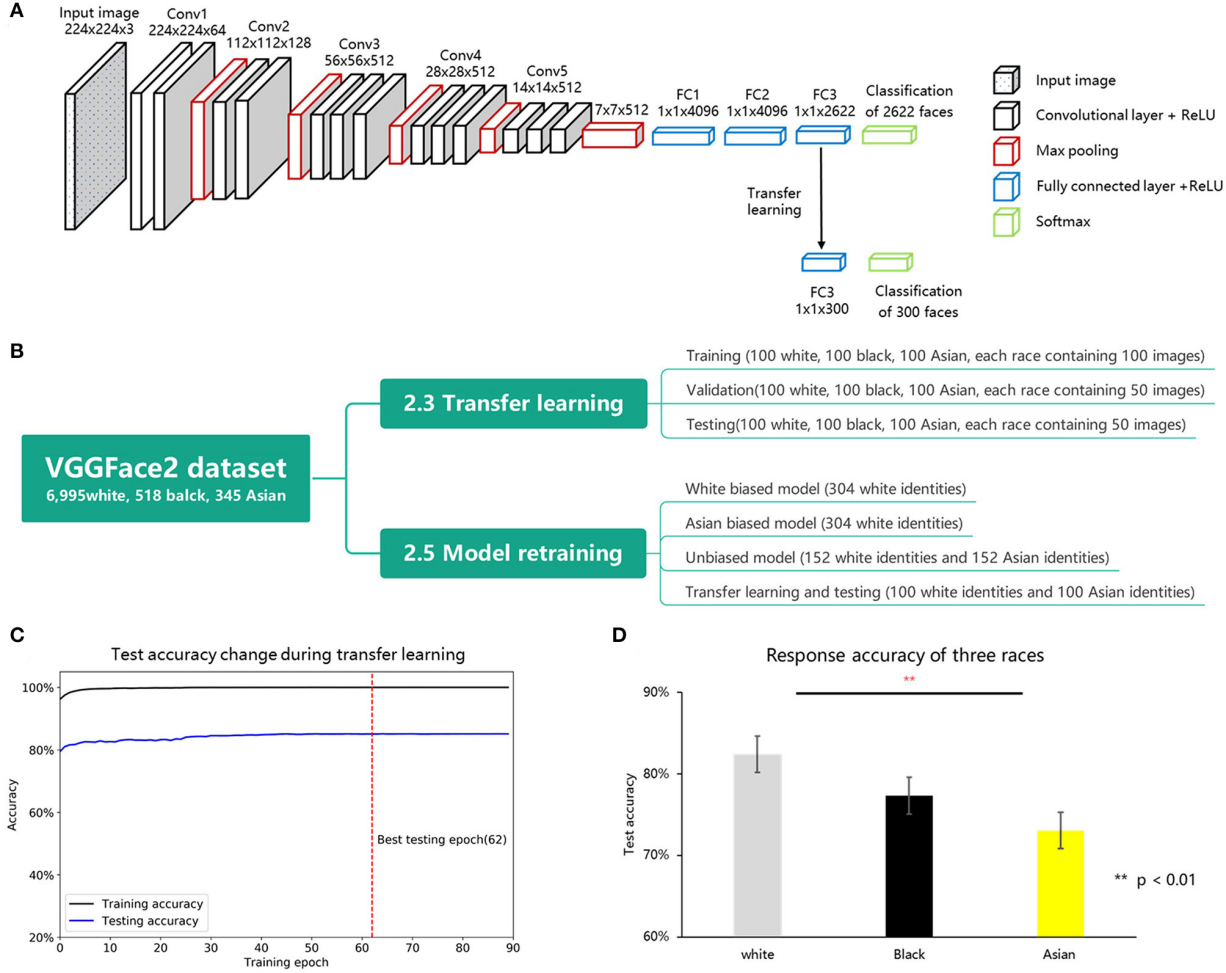
**(A)** Illustration of VGG-Face's architecture used in this study. The model comprised five convolutional blocks (conv1-conv5) and three fully connected layers (FC1-FC3). **(B)** Data organization of transfer learning and model retraining. **(C)** The change of test accuracy during VGG-Face transfer learning. The x axis represents training accuracy, and the y axis represents training epochs. The black and blue line represent training and validation accuracy changes during model training separately. **(D)** Identification accuracy of the VGG-Face on white, black, and Asian faces.

To address the aforementioned question, the current study chose a typical DCNN, VGG-Face (Figure 1A), which is widely used for face recognition (Parkhi et al., 2015). We first examined whether there was a similar ORE in VGG-Face and explored its face representation space using MDS theory. First, we manipulated the ratio of face images of different races to examine whether the ORE in the VGG-Face changed as a function of the frequencies of encountered races (Chiroro and Valentine, 1995). Secondly, we examined whether frequent interaction with one race led to sparser distribution (i.e., high distinctiveness) in VGG-Face's representation space. Thirdly, we explored whether the difference in representation led to the ORE.

## Materials and Methods

### Convolutional Neural Network Model

In this study, a well-known deep neural network, VGG-Face (available in http://www.robots.ox.ac.uk/~albanie/pytorch-models.html) was used for model testing, model retraining, and model activation extraction (Parkhi et al., 2015). An illustration of the VGG-Face architecture is shown in Figure 1A. This framework consists of five groups of convolutional layers and three fully connected layers, with 16 layers in total. Each convolutional layer comprises some convolution operators, followed by a non-linear rectification layer, such as ReLU and max pooling. The input images (for example, 3 × 224 × 224 pixels color image) are transferred into 2,622 representational units, each corresponding to a unit of the last fully connected layer (FC3), representing a certain identity.

### Face Stimuli

The VGG-Face was originally trained for face identification tasks with the VGGFace dataset (including 2,622 identities in total, with 2,271 downloadable identities).

As shown in Figure 1B, to test the performance of the VGG-Face on three races, 300 different identities were selected from another face dataset, VGGFace2 (Cao et al., 2018). Face images that were present in both the VGGFace and VGGFace2 datasets were excluded (see https://github.com/JinhuaTian/DCNN_other_race_effect/tree/master/face_materials for details). We classified the remaining 8,250 identities into four groups: white (6,995 identities), black (518 identities), Asian (345 identities), and other races (392 identities). Three hundred identities were randomly selected from the first three groups (100 identities for each race) and separated into in-house transferring learning (300 identities, each containing 100 images), validating (300 identities, each containing 50 images), and testing (300 identities, each containing 50 images) datasets. These three datasets contained the same identities but with different face exemplars; therefore, biased responses were unlikely to be introduced at the phase of transferring learning. Note that the dataset for transferring learning, validating, and testing was not overlapped with the dataset used for pre-training the network. We performed the transfer learning on the VGG-Face with the transfer learning dataset, validated the model with the validating dataset, and finally used the testing dataset to measure the identification accuracy of three different races. To confirm the reproducibility of our results, we sampled the other two datasets for transfer learning (detailed information is provided in the Supplementary Material 1.3).

### Transfer Learning

We tested the identification performance of VGG-Face with new identities using transfer learning (Yosinski et al., 2014), which trains a pre-trained network with another small set of related stimuli. Transfer learning was performed on the pre-trained VGG-Face with the in-house training set. We replaced the last FC layer (the third fully connected layer, FC3, containing 2,662 units) of the VGG-Face with another fully connected layer containing 300 units (each representing a unique face identity used in training and testing procedures). Subsequently, we froze the parameters prior to the classification layer (FC3) and trained the FC3 using the training dataset. Detailed training parameters were obtained from a previous study (Krizhevsky, 2014). All networks are trained for face identification using the cross-entropy loss function with a stochastic gradient descent (SGD) optimizer (initial learning rate = 0.01, momentum = 0.9). Images were normalized to the same luminance (mean = [0.485, 0.456, 0.406], SD = [0.229, 0.224, 0.225]) and resized to the 3 × 224 × 224 pixels. Data argumentation used 15° random rotation and a 50% chance of horizontal flip. All models were trained for 90 epochs, and the learning rate decayed 250^−1/3^ (≈ 0.159) after every 23 epochs (1/4 training epochs). To achieve optimal training accuracy and prevent overfitting, we saved the best model, which had the highest validating accuracy during training. The training procedure is shown in Figure 1C. After transfer learning, this network (the best model) was tested using the testing dataset. The performance difference between the three races was analyzed using a repeated-measures analysis of variance (ANOVA).

### Model Retraining

According to human contact theory, low interracial interactions are the main cause of ORE. For a DCNN, biased training data may lead to biased performance. To examine this hypothesis, we further retrained the VGG-Face using two “biased” face sets and one matched face set, and then tested whether these models showed a face bias. The training face sets were composed of different numbers of Asian and white faces. The different composition of Asian and white faces simulates the “white biased,” “Asian biased,” and “unbiased” datasets.

#### Retraining Materials

All images used for model retraining and validating were selected from the VGGFace2 datasets. We selected 404 Asian identities and 404 white identities for model training and testing. For the white-biased model, we randomly selected 304 white identities out of 404 identities for model training. For the Asian-biased model, we randomly selected 304 Asian identities out of 404 identities for model training. For unbiased model training, we selected 152 Asian and 152 white identities. The training datasets were further separated into training and validation sets. We selected 30 of each identity (15,000 images in total) as the validation dataset, and the remaining faces (109,450 images for the Asian biased model, 103,745 images for the white biased model, and 105,781 images for the unbiased model) were used for model training. Two hundred other identities (100 identities for each race) were selected for transfer learning and testing.

#### Retraining Procedure

Recent studies have shown that the softmax loss function in VGG-Face lacks the power of discrimination (Cao et al., 2020), and therefore may result in the ORE observed in the network. To rule out this possibility, we re-trained VGG-Face with new loss functions, such as focal loss (Lin et al., 2017) and Arcface (Deng et al., 2019), which are designed to solve the simple hard example imbalance or long-tailed problem caused by imbalanced training data. We used the same VGG-Face framework as the pre-trained model. All networks were trained for face identification with a stochastic gradient descent (SGD) optimizer (initial learning rate = 0.01, momentum = 0.9). Images were normalized to the same luminance (mean = [0.485, 0.456, 0.406], SD = [0.229, 0.224, 0.225]) and resized to 3 × 224 × 224 pixels. Data argumentation used 15° random rotation and a 50% chance of horizontal flip. All models were trained for 90 epochs, and the learning rate decayed 250^−1/3^ (≈ 0.159) after every 23 epochs (1/4 training epochs). To achieve optimal training accuracy and prevent overfitting, we saved the best model, which had the highest validating accuracy during training. The saved model was used for further model testing using the testing dataset.

### Face Representation Difference of Three Races in VGG

To explore the representation pattern of different races in VGG-Face, we further analyzed the face representation difference. It has been suggested that activation responses of the layer prior to the final classification layer (the second fully connected layer: FC2) is a typical representation of each face in DCNNs (O'toole et al., 2018). Thus, we extracted the activation responses in the FC2 layer for all the testing faces using an in-house Python package, namely, DNNBrain (Chen et al., 2020) with the PyTorch framework (Paszke et al., 2019).

To describe the distinctness of each race group, we used three measurements to describe the distribution of face space. First, we applied the representation similarity analysis to obtain the representational dissimilarity correlation matrix (RDM) of three race faces with FC2 activation. To further explore the representation difference between the three races, we used the in-group similarity to describe representation variance within a race group. The in-group similarity was calculated as the averaged Pearson correlation of a certain identity with other identities of the same race. Specifically, a face with larger in-group similarity indicated smaller representation distinctiveness. That is, the larger the distinctiveness, the better the performance in discriminating identities.

Next, we used FC2 activation to construct the face space describing the distribution of different faces. Valentine and Endo (1992) assume the face space to be an n-dimensional space; a face is represented as a point localized in the space. The axes of the space represent dimensions to discriminate faces. According to this hypothesis, we used the average activation of all faces as the possible center coordinates of this face space. Thus, we computed the Euclidean distance of the averaged activation from each face to all averaged face activations as a measurement of face distinctiveness. A face with a larger Euclidean distance indicated larger representation distinctiveness. The activation differences in the three races were also analyzed using a one-way ANOVA.

### Face Representation Visualization

For a better visualization of the representation of the face space, we used the t-SNE (t-distributed stochastic neighbor embedding, t-SNE) method to reduce face representation dimensions and visualize the activation distribution. The t-SNE starts by converting the high-dimensional Euclidean distances between data points into conditional probabilities that represent similarities (Van Der Maaten and Hinton, 2008). We used the t-SNE to squeeze the activation vectors (2,622 units) of each face's activation into two dimensions and plotted these conditional probabilities on a two-dimensional coordinate for visualization. The t-SNE was performed using default parameters (learning rate = 200, iteration = 1,000).

### Correlation Between Face Representation and Identification Performance

To explore whether VGG-Face activation and its performance were correlated, we computed the Spearman correlation as well as the Pearson correlation between the in-group similarity and Euclidean distance with face identification accuracy of the VGG-Face.

## Results

First, we used transfer learning to examine race bias in the VGG-Face. The average accuracy of all identities was 77.6%, significantly higher than the stochastic probability (0.33%), indicating the success of transfer learning. A one-way ANOVA showed a significant main effect of race (*F*_2, 297_ = 8.762, *p* < 0.001, $\eta_{p}^{2}$ = 0.056), with white faces being identified significantly better than Asian faces (*p* < 0.001, *d*′ = 0.545) and marginally significantly better than black (*p* = 0.071, *d*′ = 0.353) faces (Figure 1D). No significant difference was found in accuracy between the identification of black and Asian faces (*p* = 0.176, *d*′ = 0.255).

To verify face selection bias in VGG network training, we classified the available VGGFace dataset into four groups, namely, white (1,984 identities, 87.2%), black (211 identities, 9.7%), Asian (52 identities, 2.3%), and other races (brown or mixed race, 24 identities, 1.1%). As faces in the dataset were overwhelmingly white, the better identification accuracy for white faces suggested that the ORE also existed in the VGG-Face.

**Figure 2:**
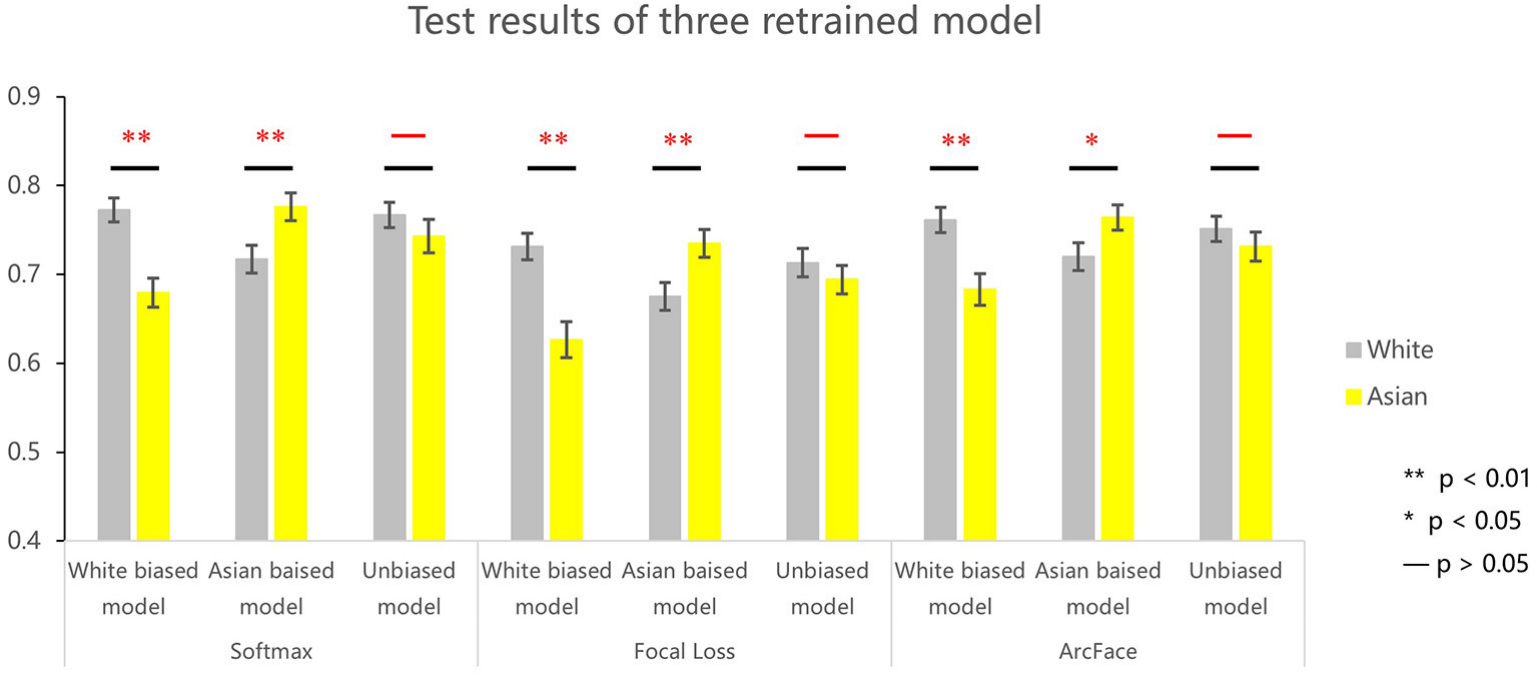
Identification performance of three retrained VGG networks (i.e., white biased model, Asian biased model, and unbiased model) using softmax, focal loss, and Arcface. **p < 0.01; *p < 0.05; –, not significant.

A direct test on whether the ORE observed in the VGG-Face resulted from the imbalance of races present in the dataset was to manipulate the ratio of the number of faces of each race. To do this, we retrained the VGG network using white-biased (white vs. Asian: 100 vs. 0%), Asian biased (0 vs. 100%), and unbiased (50 vs. 50%) datasets, respectively. As shown in Figure 2, the three DCNNs showed different patterns of ORE. For the DCNN trained with the white-biased dataset, white faces were identified significantly better than Asian faces (softmax: *t*_198_ =3.934, *p* < 0.001, *d*′ = 0.562; focal loss: *t*_198_ =4.203, *p* < 0.001, *d*′ = 0.617; Arcface: *t*_198_ = 3.405, *p* < 0.001, *d*′ = 0.486). In contrast, in the Asian-biased DCNN, Asian faces were identified better than white faces (softmax: *t*_198_ = 2.693, *p* = 0.008, *d*′ = 0.381; focal loss: *t*_198_ = 2.689, *p* = 0.008, *d*′ = 0.382; Arcface: *t*_198_ = 2.0880., *p* = 0.038, *d*′ = 0.296). Finally, no ORE was found in the unbiased DCNN (softmax: *t*_198_ = 1.135, *p* = 0.258, *d*′ = 0.161; Focal loss: *t*_198_ = 0.905, *p* = 0.367, *d*′ = 0.132). Taken together, the ORE observed in the VGG-Face resulted from unbalanced experiences with different numbers of faces per race during model training.

**Figure 3:**
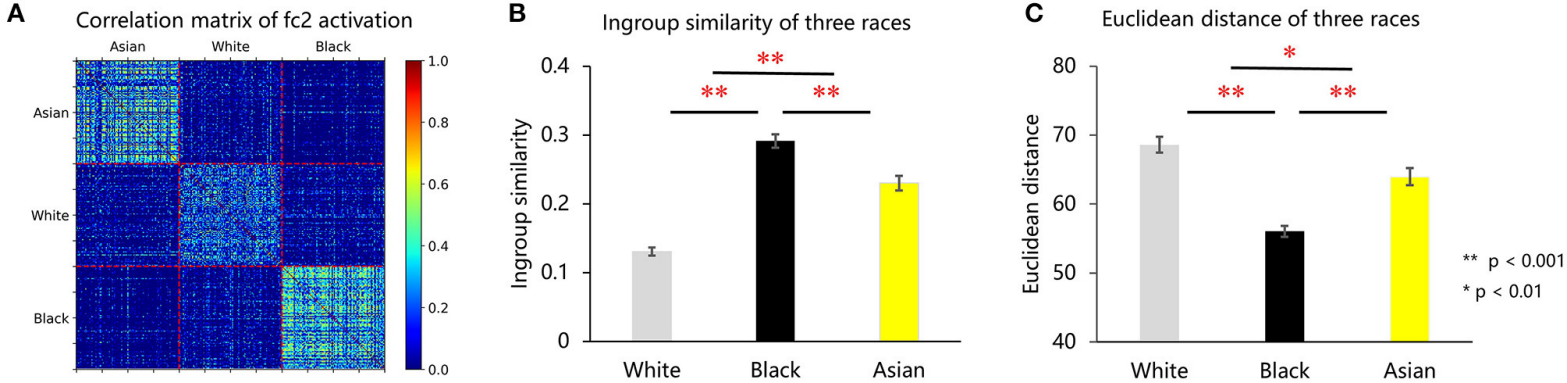
**(A)** VGG-Face FC2 activation correlation matrix of Asian, white, and black faces. **(B)** Face distinctiveness of white, black, and Asian faces measured using in-group similarity. **(C)** Face distinctiveness of white, black, and Asian faces measured using face Euclidean distance.

How do unbalanced experiences shape the internal representation of faces in the VGG-Face? To address this question, we calculated the correlations between the representations of faces, which were indexed by the activations in the FC2 layer, and then constructed a correlation matrix consisting of Asian, white, and black faces (Figure 3A). A direct observation of Figure 3A revealed that faces of each race were grouped into one cluster; that is, the representations for faces were more similar within a race than between races, suggesting that faces from the same race were grouped together in the multidimensional space. Importantly, the representational similarity of white faces was smallest, compared with Asian (*p* < 0.001, *d*′ = 1.29) and black (*p* < 0.001, *d*′ = 2.077) faces, and that of Asian faces was smaller than that of black faces (*p* < 0.001, *d*′ = 0.4) (Figure 3B). That is, the representations for white faces were the sparsest in the face space. To quantify the sparseness of the representation, we calculated the Euclidean distance of the representation of individual faces to the averaged representation of all faces. As shown in Figure 3C, the representation of white faces was localized farther from the averaged representation than that of Asian (*p* = 0.008, *d*′ = 0.386) and black (*p* < 0.001, *d*′ = 1.286) faces, and that of Asian faces was farther than that of black faces (*p* < 0.001, *d*′ = 0.773). The activation of faces in the last fully connected layer (FC3) was also extracted and analyzed, which showed a similar representational pattern as FC2 (detailed information is provided in the Supplementary Materials).

**Figure 4:**
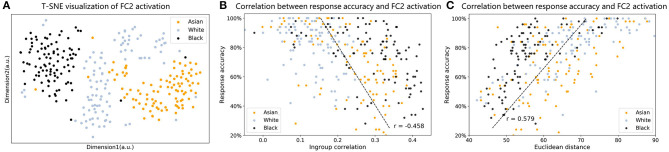
**(A)** T-SNE visualization of FC2 activation of Asian, white, and black faces. **(B)** Correlation between in-group similarity and face identification accuracy. **(C)** Correlation between face Euclidean distance to averaged face activation and face identification accuracy.

To visualize how race faces were represented in the face space, we used t-SNE to reduce multiple dimensions to two dimensions. As shown in Figure 4A, representations for each race were grouped into one cluster; however, the clusters for Asian and black faces were denser, whereas white faces were distributed more sparsely in the face space.

Finally, we explored whether the difference in sparseness of the representation was related to the ORE observed in VGG-Face. As shown in Figure 4B, the correlation analysis showed a significant negative correlation between in-group similarity and face identification accuracy (coefficient Pearson's correlation *R* = −0.458, *p* < 0.001, Spearman correlation *R* = −0.499, *p* < 0.001). As shown in Figure 4C, the correlation analysis showed a significant positive correlation between Euclidean distance and face identification accuracy (coefficient Pearson's correlation *R* = 0.579, *p* < 0.001, Spearman correlation *R* = 0.621, *p* < 0.001). That is, if a face was represented further from the average representation, it was more accurately identified by the VGG-Face. For the VGG-Face trained by a dataset dominated by white faces, white faces on average had the largest representational distance, and they were the most likely to be identified correctly, which therefore resulted in the ORE.

## Discussion

In this study, we examined the ORE in VGG-Face. By manipulating the ratio of faces of different races in the training dataset, the results demonstrated that unbalanced datasets led to the appearance of the ORE in VGG-Face, in line with studies on humans, which have reported that visual experiences affect the identification accuracy of a particular race's face (Chiroro and Valentine, 1995; Meissner and Brigham, 2001). Importantly, the representation similarity analysis revealed that if white faces dominated the dataset, they were distributed more sparsely in the multidimensional representational space of faces in VGG-Face, resulting in better behavioral performance. On the other hand, a similar phenomenon, called “long tailed problem,” suggested that the model performs better on the head domains (i.e., high-frequency domain) than on the tail domains (i.e., low-frequency domain). The inter-class distance was usually used to distinguish the head domain from the tail domain. The head domain usually showed a larger inter-class indicator than that of the tail domain (Cao et al., 2020), which seems to be opposite to our result. In our study, we used intra-class distance (in-group similarity and in-group Euclidean distance), which was widely used to quantify the sparseness of the representation. We found the faces of the majority race were scattered more sparsely in the representational face space. This result is consistent with previous results in humans (Valentine, 1991; Valentine et al., 2016), which implied a similar mechanism. In sum, with the MDS theory in human, we provided a novel approach to understand race biases in DCNNs.

The AI ethical problem has attracted broad attention to the field of AI (Zemel et al., 2013; Zou and Schiebinger, 2018). However, the mechanism underlying AI biases is poorly understood. Our study confirmed that the ORE bias might be derived from an unbalanced training dataset. This is consistent with the contact theory (Chiroro and Valentine, 1995) in humans, according to which high-contact faces are recognized more accurately than low-contact ones. Previous studies in humans suggest that high in-group interaction leads to sparser representation (high distinctiveness) of in-group faces in face space, whereas low interaction leads to denser representation (low distinctiveness) of out-group faces (Valentine, 1991; Valentine et al., 2016). In the current study, we also found that in the representational space of VGG-Face, “own-race” faces (i.e., white faces) showed larger distinctiveness than that of “other-race” faces (i.e., Asian and black faces). Furthermore, the distinctiveness was indexed by the representational similarity of faces, which may serve as a more sensitive index than the ratio of faces in the unbalanced dataset. Therefore, before formal training, an examination of representational similarity in MDS with a portion of the training dataset may provide an estimate of the skewness of the datasets and the biased performance under current task demands.

Therefore, a more effective way of controlling AI biases may come from new algorithms that can modulate the internal representations of DCNNs. Currently, most efforts have been focused on the construction of balanced datasets and the approaches of training DCNNs, and guidelines have been advised (Gebru et al., 2018; Mitchell et al., 2019). However, it is laborious to balance datasets not only in terms of data collection, but also in terms of task demands. It might be more efficient if a revised back-propagation algorithm could minimize errors between outputs and goals and rectify differences in distinctiveness of the representation of interests. For example, in the field of natural language processing, Beutel et al. (2017) and Zhang et al. (2018) proposed a multi-task adversarial learning method to manipulate the biased representational subspace and thus mitigate the gender bias of model performance. They built a multi-head DCNN where one head was for target classification and another was for removing information about unfair attributes learned from the data. Similarly, in the field of computer vision, further studies could also explore ways to manipulate the face representational space to reduce social bias in DCNNs.

In conclusion, our study used a well-known phenomenon, the ORE, to investigate the mechanism inside DCNNs that leads to biased performance. In addition, we found a human-like multidimensional face representation in DCNN, suggesting that paradigms and theories discovered in human studies may also be helpful in identifying the underlying mechanisms of DCNNs. There are many other types of biases in AI, such as gender bias and age bias; therefore, our study invites broad investigation on these ethical problems in AI.

## Data Availability Statement

The original contributions presented in the study are publicly available. All face image materials and model training codes used in this article are provided on git-hub: https://github.com/JinhuaTian/DCNN_other_race_effect.

## Author Contributions

JL and SH designed the research. JT and HX collected and analyzed the data. JT wrote the manuscript with input from JL and SH. All authors reviewed and commented on this manuscript.

## Conflict of Interest

The authors declare that the research was conducted in the absence of any commercial or financial relationships that could be construed as a potential conflict of interest.
