# Supplementary material for: Multidimensional Face Representation in a Deep Convolutional Neural Network Reveals the Mechanism Underlying AI Racism
Source: Front Comput Neurosci. 2021 Mar 10;15:620281. doi: 10.3389/fncom.2021.620281 (PMC7987832; doi:10.3389/fncom.2021.620281)
Supplement: Supplementary file 1 [file Data_Sheet_1.docx]

Supplementary Material

## Face-race representation difference of three races in FC3

As for fc3, One-way ANOVAs results showed significant main race effect in in-group similarity ($F_{2, 297}$ = 121.327, *p* < 0.001, $\eta_{p}^{2}$ = 0.450). As shown in Figure 1A, pairwise analysis (with Bonferroni correction) indicated that white faces showed significantly smaller in-group similarity (larger distinctiveness) than Asian (*p* < 0.001,$d^{'}$ = 0.863) and black (*p* < 0.001, $d^{'}$ = 2.590) faces, and Asian faces showed smaller in-group similarity than black faces (*p* < 0.001, $d^{'}$ = 1.222). This result indicated that white faces showed less in-group representational similarity than both Asian and black faces in VGG network.

Moreover, one-way ANOVAs results also showed a significant main race effect in both Euclidean distance ($F_{2, 297}$ = 36.994, $\eta_{p}^{2}$ = 0.199, *p* < 0.001). As shown in Figure 1B, pairwise comparison (with Bonferroni correction) showed that white faces were localized farther than Asian (*p* < 0.001, $d^{'}$ = 0.528) and black (*p* < 0.001, $d^{'}$ = 1.222) faces, and Asian faces were localized farther than black faces (*p* < 0.001, $d^{'}$ = 0.763).

These results indicated that fc3 showed similar representational pattern as fc2. That is, white faces showed larger distinctiveness (smaller in-group similarity and larger Euclidean distance) than Asian and black faces.

## Correlation between face representation and identification performance in FC3

For FC3 activation, As shown in Figure 1C, correlation analysis showed a significant negative correlation (coefficient Pearson’s R = -0.368, *p* < 0.001, coefficient Spearman correlation R = -0.420, *p* < 0.001) between in-group similarity and face identification accuracy. As shown in Figure 1D, correlation analysis also showed a significant positive correlation between Euclidean distance (coefficient Pearson’s correlation R = 0.451, *p* < 0.001, Spearman correlation R = 0.478, *p* < 0.001) and VGGFACE identification accuracy. FC3 results showed similar activation-performance correlation-ship as FC2, which indicated that a face with higher identification accuracy (larger face distinctiveness) showed smaller in-group representational similarity with other faces, and localized farther in the face space.


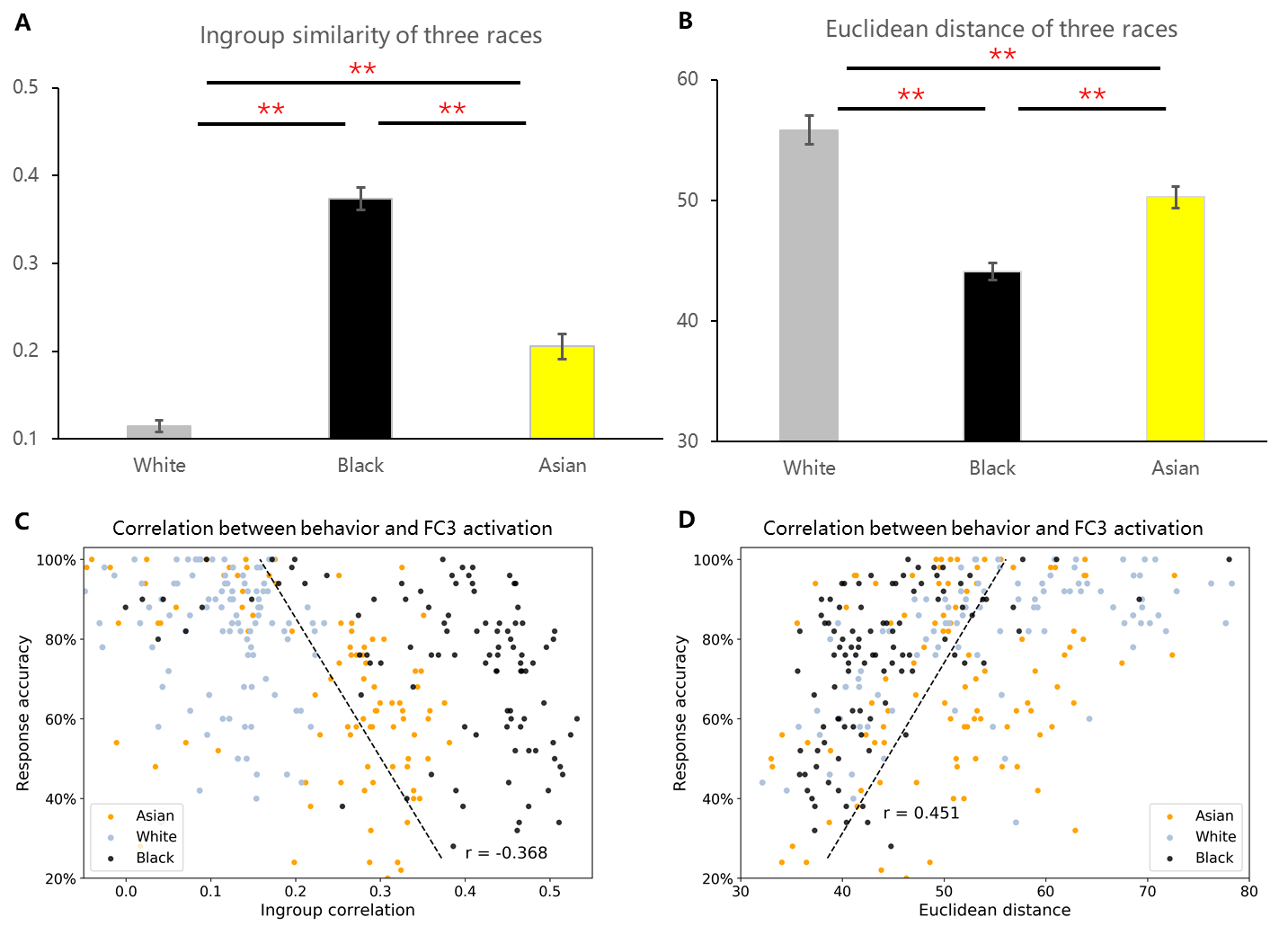


**FIGURE 1. | (A)** Face distinctiveness of Asian, white, and black faces measured using in-group similarity. **(B)** Face distinctiveness of Asian, white, and black faces measured using face Euclidean distance to all averaged face activation. **(C)** correlation between face distinctiveness and VGG face identification accuracy in FC3. **(D)** correlation between Face Euclidean distance and VGG face identification accuracy in FC3.

## Validation of the model testing and representation results.

To further validate the generalization of our results, we sampled other two datasets and repeated our data analysis (transfer learning and face representation analysis).

### Validation face stimuli

We sampled other two transfer learning materials and test their performance using similar procedure. Sample 1 contains 100 Asian identities, 100 white identities and 100 black identities, and we separated this sample into in-house training (300 identities, each containing 100 images), validating (300 identities, each containing 50 images), and testing (300 identities, each containing 50 images). Sample 2 contains 100 Asian identities, 100 white identities and 100 black identities, and we separated this sample into in-house training (300 identities, each containing 120 images), validating (300 identities, each containing 50 images), and testing (300 identities, each containing 50 images).

### transfer learning results

For sample 2, as shown in Fig 2A, A one-way ANOVA showed a significant main effect of race ($F_{2, 297}$ = 42.127, *p* < 0.001, $\eta_{p}^{2}$ = 0.221), with white faces being identified significantly better than Asian faces (*p* < 0.001, *d^’^* = 1.247) and significantly better than black (*p* < 0.001, *d^’^* *=* 0.76) faces (Figure 1C). For sample 3, as shown in Fig 2B, A one-way ANOVA showed a significant main effect of race ($F_{2, 297}$ = 42.127, *p* < 0.001, $\eta_{p}^{2}$ = 0.221), with white faces being identified significantly better than Asian faces (*p* < 0.001, *d^’^* = 1.17) and significantly better than black (*p* = 0.002, *d^’^* *=* 0.643) faces (Figure 1C).


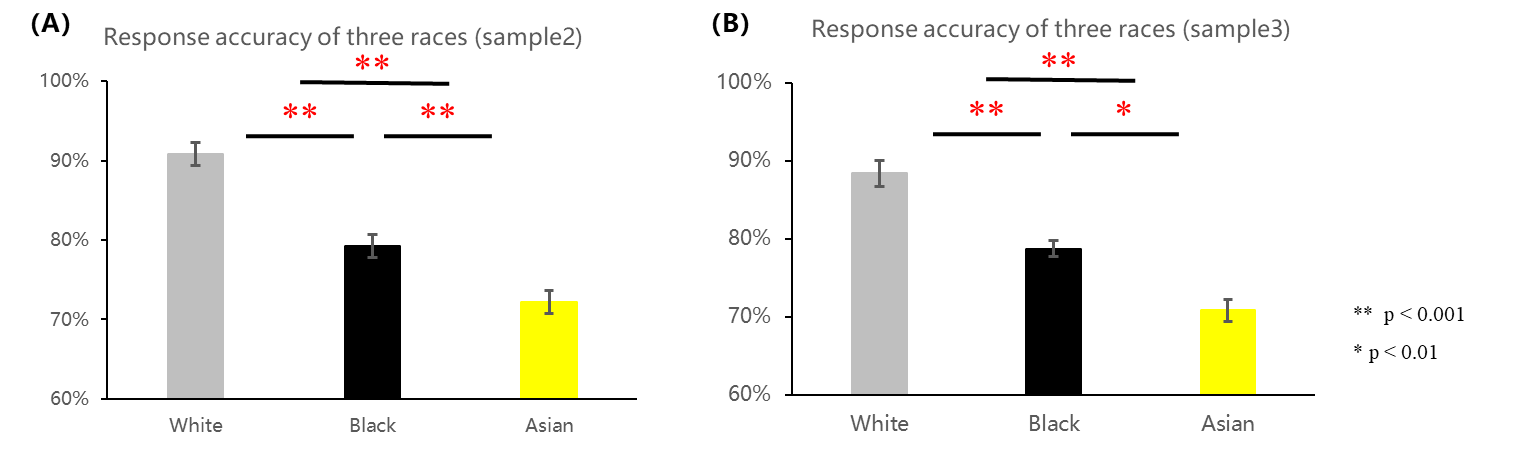


**FIGURE 2 | (A)**, **(B)** Identification accuracy of the VGG-Face on white, black, and Asian faces of sample 2 and sample 3

### Face representation difference of three races in VGG

For sample 2, the representational similarity of white faces was smallest, compared with Asian (*p* < 0.001, *d^’^* = 1.534) and black (*p* < 0.001, *d^’^* = 1.353) faces, and that of Asian faces and that of black faces showed no significant difference (*p* > 0.5, *d^’^* = 0.184) (Figure 3A). As shown in Figure 3C, Euclidean distance results showed that the representation of white faces was localized farther from the averaged representation than that of Asian (*p* < 0.001, *d^’^* = 1.221) and black (*p* < 0.001, *d^’^* = 1.249) faces, and that of Asian faces and that of black faces showed no significant difference (*p* > 0.5, *d^’^* = 0.02).

For sample 3, the representational similarity of white faces was also the smallest, compared with Asian (*p* < 0.001, *d^’^* = 1.514) and black (*p* < 0.001, *d^’^* = 2.147) faces, and that of Asian faces and that of black faces showed no significant difference (*p* > 0.5, *d^’^* = 0.086) (Figure 3B). As shown in Figure 3D, Euclidean distance results showed that the representation of white faces was localized farther from the averaged representation than that of Asian (*p* < 0.001, *d^’^* = 1.562) and black (*p* < 0.001, *d^’^* = 1.455) faces, and that of Asian faces and that of black faces showed no significant difference (*p* > 0.5, *d^’^* = 0.113).

These results validated that white faces showed larger distinctiveness (smaller in-group similarity and larger Euclidean distance) than Asian and black faces.


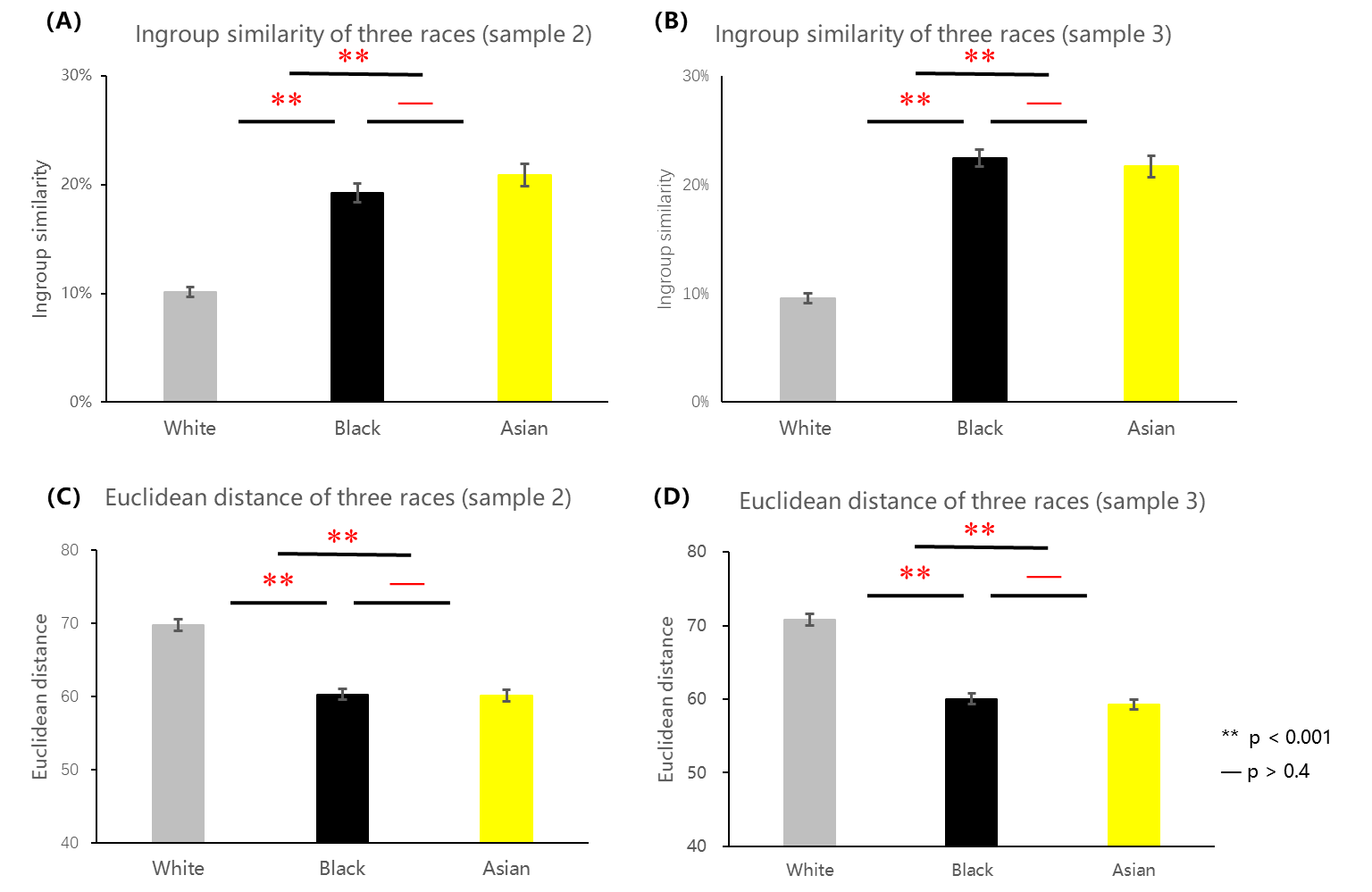


**FIGURE 3 | (A)**, **(B)** Face distinctiveness of white, black, and Asian faces measured using in-group similarity. **(C)**, **(D)** Face distinctiveness of white, black, and Asian faces measured using face Euclidean distance.

### Correlation between face representation and identification performance

As shown in Figure 4A, the correlation analysis of sample 2 showed a significant negative correlation between in-group similarity and face identification accuracy (coefficient Pearson’s correlation R = -0.524, *p* < 0.001, Spearman correlation R = -0.572, *p* < 0.001). As shown in Figure 4B, the correlation analysis showed a significant positive correlation between Euclidean distance and face identification accuracy (coefficient Pearson’s correlation R = 0.703, *p* < 0.001, Spearman correlation R = 0.766, *p* < 0.001).

As shown in Figure 4C, the correlation analysis of sample 3 showed a significant negative correlation between in-group similarity and face identification accuracy (coefficient Pearson’s correlation R = -0.556, *p* < 0.001, Spearman correlation R = -0.580, *p* < 0.001). As shown in Figure 4D, the correlation analysis showed a significant positive correlation between Euclidean distance and face identification accuracy (coefficient Pearson’s R = 0.701, *p* < 0.001, coefficient Spearman correlation R = 0.775, *p* < 0.001).

These results proved that if a face was represented further from the average representation, it was more accurately identified by the VGG-Face.


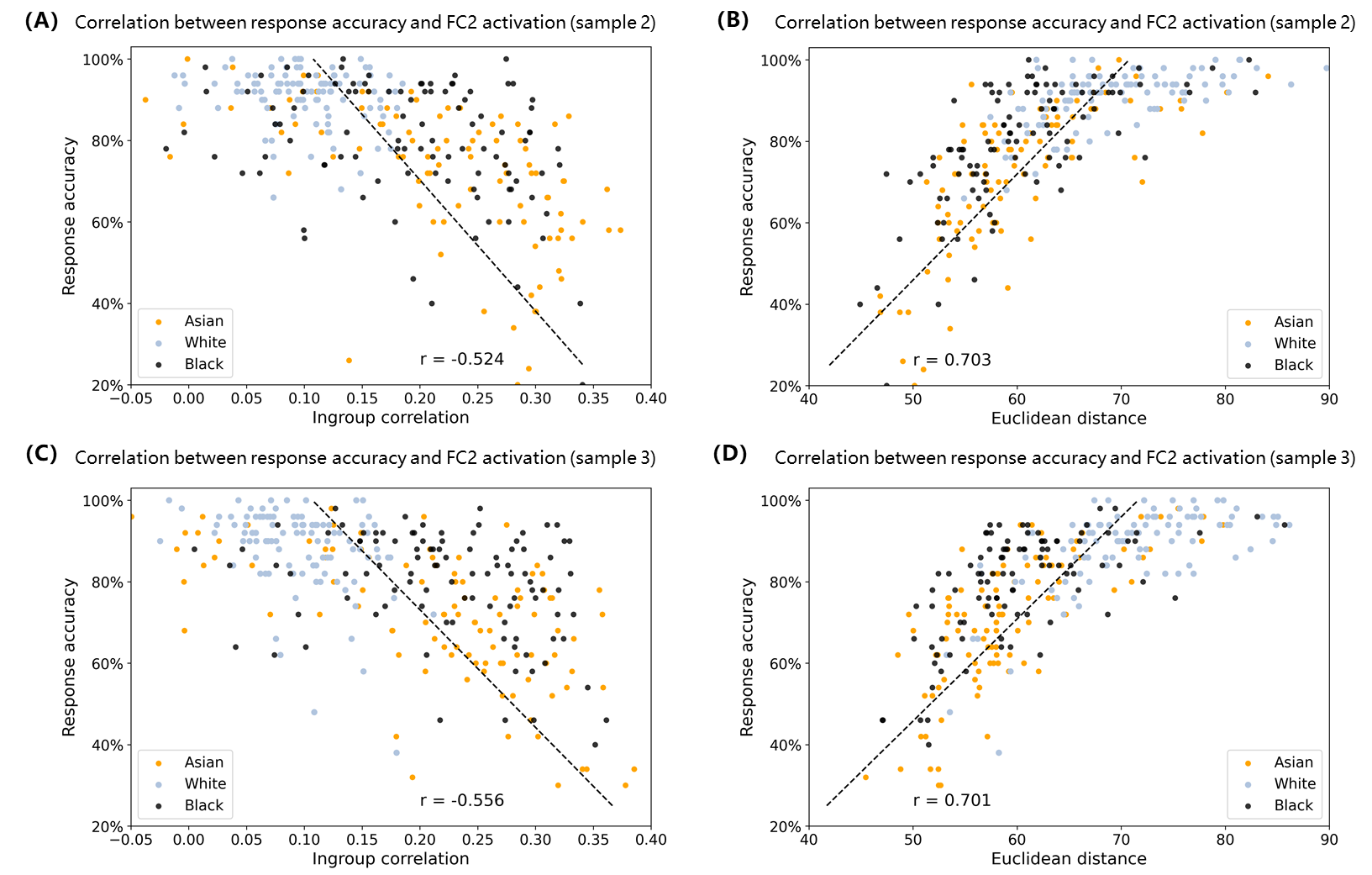


**FIGURE 4 (A)** As for sample 2, correlation between in-group similarity and face identification accuracy. **(B)** As for sample 2, correlation between face Euclidean distance to averaged face activation and face identification accuracy. **(C)** As for sample 3, correlation between in-group similarity and face identification accuracy. **(D)** As for sample 3, correlation between face Euclidean distance to averaged face activation and face identification accuracy.
